# Harnessing Data Augmentation and Normalization Preprocessing to Improve the Performance of Chemical Reaction Predictions of Data-Driven Model

**DOI:** 10.3390/polym15092224

**Published:** 2023-05-08

**Authors:** Boyu Zhang, Jiaping Lin, Lei Du, Liangshun Zhang

**Affiliations:** Shanghai Key Laboratory of Advanced Polymeric Materials, School of Materials Science and Engineering, East China University of Science and Technology, Shanghai 200237, China

**Keywords:** chemical reaction, retrosynthesis, data augmentation, machine learning, molecular transformer model

## Abstract

As a template-free, data-driven methodology, the molecular transformer model provides an alternative by which to predict the outcome of chemical reactions and design the route of the retrosynthetic plane in the field of organic synthesis and polymer chemistry. However, in consideration of the small datasets of chemical reactions, the data-driven model suffers from the difficulty of low accuracy in the prediction tasks of chemical reactions. In this contribution, we integrate the molecular transformer model with the strategies of data augmentation and normalization preprocessing to accomplish the three tasks of chemical reactions, including the forward predictions of chemical reactions, and single-step retrosynthetic predictions with and without the reaction classes. It is clearly demonstrated that the prediction accuracy of the molecular transformer model can be significantly raised by the use of proposed strategies for the three tasks of chemical reactions. Notably, after the introduction of the 40-level data augmentation and normalization preprocessing, the top-1 accuracy of the forward prediction increases markedly from 71.6% to 84.2% and the top-1 accuracy of the single-step retrosynthetic prediction with additional reaction class increases from 53.2% to 63.4%. Furthermore, it is found that the superior performance of the data-driven model originates from the correction of the grammatical errors of the SMILES strings, especially for the case of the reaction classes with small datasets.

## 1. Introduction

The prediction of chemical reactions and the design of a synthetic route are the key steps involved in the problem-solving tasks of organic synthesis and polymer chemistry [1], which is used to create new molecules from simple commercially available compounds. Because of its complexity, organic synthesis is believed to be one of the main bottlenecks in the preparation of organic molecular materials, as well as the discovery of novel medicines [2,3,4]. Accurate models to predict the output of chemical reactions could boost chemists’ productivity by reducing the number of experiments to be performed [1,5,6,7,8,9,10,11,12,13].

**Figure 1:**
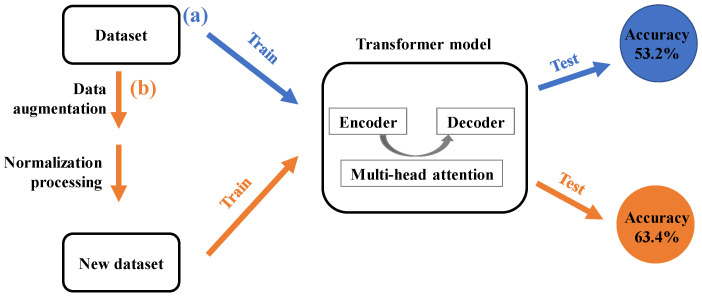
Schematic diagram of the workflow for the chemical reaction predictions. (**a**) Original workflow: the transformer model is used to predict the consequence of chemical reactions. (**b**) Our proposed workflow: the strategies of data augmentation and normalization preprocessing are incorporated into the transformer model in order to improve the prediction accuracy.

Machine learning has long been presented in the chemical domains, tackling the challenges associated with structure–activity relationship predictions [14,15,16,17], virtual screening [18,19,20,21] and quantum chemistry [22,23,24,25]. Enabled by algorithmic advances in deep learning and the availability of reaction datasets, the methods used to predict chemical reactions have advanced in recent years [26,27,28,29,30,31]. In particular, the chemical compounds can be equivalently expressed as text sequences, such as the simplified molecular-input line-entry system (SMILES) [32]. The tasks of chemical reaction prediction can be regarded as a problem of translating natural language in machine learning, where the objective is to map a text sequence of reactant compounds to a text sequence of product compounds. In order to achieve the tasks, a neural sequence-to-sequence model was developed to realize the completely end-to-end prediction of forward reaction without the need of any atom-mapped reaction instances [28]. Unfortunately, the model does not enhance accuracy significantly over the rule-based method and provides a large number of chemically erroneous results. Recently, the transformer architecture has demonstrated the benefits of machine translation [33]. It is only dependent on the self-attention mechanism, allowing for the extraction of both local and global characteristics regardless of the separation between the input and output sequences. For instance, Schwaller applied the transformer model to predict the consequences of chemical reactions and obtained cutting-edge findings (Figure 1a) [34].

Although the transformer model can learn the chemical knowledge from data sets without human intervention, the prediction accuracy of these methods is relatively low due to a small, non-normalized dataset [31]. As an important tool in artificial intelligence (AI), data augmentation, which provides the same entity with numerous representations, can be utilized to overcome the restriction of limited amounts of data. More recent works have demonstrated the successful implementation of data augmentation in a variety of neural networks [35,36,37]. A chemical reaction can be represented by several strings via the SMILES enumeration, and the data-augmented model can learn more about a reaction by employing a batch of randomly chosen SMILES strings. Therefore, in conjunction with the normalization preprocessing of the SMILES strings, the data augmentation by the SMILES enumeration provides a clue regarding the promotion of the performance of the transformer model in order to predict chemical reactions. 

In this contribution, we incorporate the data augmentation and normalization preprocessing strategies used by the SMILES strings into the transformer architectures, which are schematically illustrated in Figure 1b. In comparison to the molecular transformer model without data augmentation and normalization preprocessing, our proposed strategies have the ability to significantly improve the accuracy of the model. In particular, the improved model achieves excellent results in both forward and retrosynthetic predictions, with top-1 accuracies of 84.2% and 63.4%, respectively. Furthermore, the grammatically invalid rate of the predicted results is analyzed.

## 2. Dataset and Methods

### 2.1. Dataset

**Figure 2:**
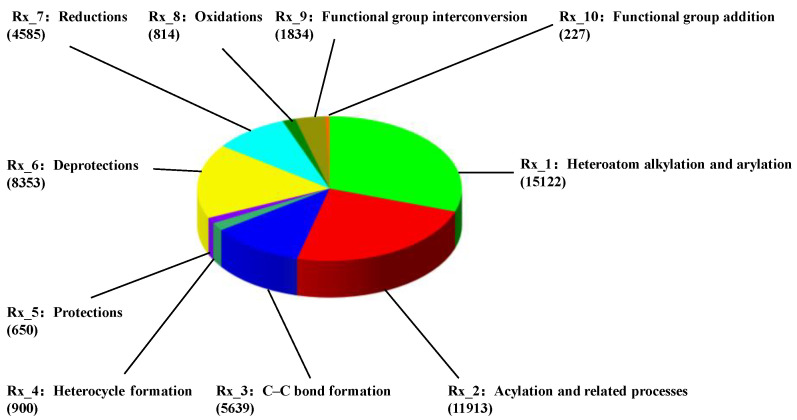
Distribution of reaction classes within the USPTO-50K. Rx_n denotes the reaction class as well as the corresponding name and number listed below.

The reaction data for the training of model were obtained from Lowe’s work [38]. The dataset we utilized contains 50,000 reaction items (designated as USPTO-50K), which is a common benchmark in the field of the AI-assisted prediction of chemical reactions. Inspired by Liu et al. [31], the items in the dataset were divided into 10 reaction classes. Figure 2 shows the reaction class (denoted as Rx_n), reaction name and the corresponding number in the dataset of USPTO-50K. In comparison with the cases of heteroatom alkylation and arylation (Rx_1), acylation and related processes (Rx_2), and deprotections (Rx_6), the data of heterocycle formation (Rx_4), protections (Rx_5), oxidations (Rx_8) and functional group addition (Rx_10) are extremely scarce in the dataset of USPTO-50K. It should be mentioned that a larger dataset is required to achieve a reliable model for the prediction of chemical reactions, especially for the transformer-based architecture of AI-assisted models. 

### 2.2. Data Preprocessing

**Figure 3:**
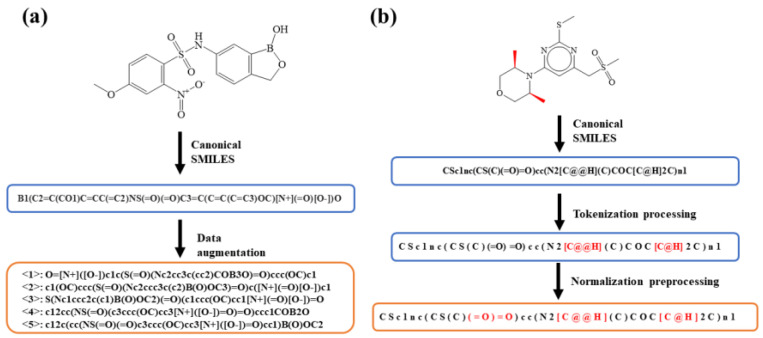
(**a**) An example of data augmentation by the SMILES enumeration. All the SMILES strings represent the same compound and the canonical SMILESs are listed in the dataset of USPTO-50K. (**b**) An example of normalization preprocessing for the input SMILES. The normalization preprocessing splits the SMILES into a string of characters.

Data augmentation can provide a more detailed description of molecules by enumerating various SMILES strings, and can enable the model to obtain more unique data points from the data. As a promising method of data augmentation in cheminformatics, the SMILES enumeration has the ability to expand the amount of SMILES strings for each molecule. It has been demonstrated that the AI-assisted models trained by a batch of random SMILES strings (i.e., data augmentation) outperform the canonicalization process [37,38,39,40,41], especially when the training set is small. The data augmentation strategy is achieved by the SMILES enumeration on the basis of the chemical information library RDKit. The atomic order of molecules can be randomly selected in the RDKit molar format, where different atomic orders result in different SMILES strings. As schematically illustrated in Figure 3a, the starting atom and the direction of the molecular graph are randomly chosen in the SMILES enumeration (also known as the “random” SMILES), resulting in *N*-level data augmentation. Note that the strategy of data augmentation is only performed on the training dataset via the method of SMILES enumeration.

Generally, the SMILES strings are tokenized in order to obtain a token-based SMILES before it is input into the transformer model. However, the token-based SMILES strings are not suitable for the transformer model, because they are not total charactered. Therefore, before inputting the data into the molecular transformer model, the character-based method is used to normalize the SMILES strings of the augmented dataset. Figure 3b shows an example of the normalization preprocessing for the SMILES string of molecules, which splits the reactants and products into characters. The normalized strings act as the input of the molecular transformer model. 

### 2.3. Model

In this study, the molecular transformer model was used to predict the chemical reactions of organic compounds. Using the molecular transformer model, we carried out three tasks: forward predictions of chemical reactions and single-step retrosynthesis with and without additional reaction classes. In the task of forward predictions, the reactants acting as the input of the AI-assisted model were used to predict their output, namely the products of chemical reactions. In contrast, the retrosynthetic tasks that corresponded to the prediction of reactants from the given products with and without additional reaction classes acted as the input of the model. Following previous works [31,42], we used accuracy as the evaluation metric. The reported accuracies describe the percentage of correct reactions. A reaction was counted as correct only if the predicted products/reactants exactly matched the chemical compounds reported in the literature after the canonicalization. Details of the architecture of the model are provided in ref [33,34].

The USPTO-50K dataset was partitioned into a 45K/5K train/test split. We used a beam search with a size of 10 to decode the top-*k* outputs. This work was built on the Open-NMT-PyTorch packages [43]. The data augmentation of the SMILES enumeration was performed with a Python script (v3.7) utilizing the RDkit (v2019.03).

## 3. Results and Discussion

In conjunction with the data augmentation and normalization preprocessing of the USPTO-50K dataset, the molecular transformer model was used to accomplish the three tasks of chemical reactions, including the forward predictions of chemical reactions, and single-step retrosynthetic predictions with and without the reaction classes.

### 3.1. Model Performance on Forward Predictions of Chemical Reactions

In the task of forward predicting chemical reactions, the input and output of the molecular transformer model are the reactants and products, respectively. To verify the effectiveness of data augmentation and normalization preprocessing, we implemented a series of training tasks with various *N*-levels of SMILES enumeration and character-based preprocessing to evaluate the performance of the molecular transformer model. *N*-level data augmentation corresponds to each chemical compound with *N* different SMILES representations. Note that the 1-level corresponds to the case of the original dataset of USPTO-50K.

**Figure 4:**
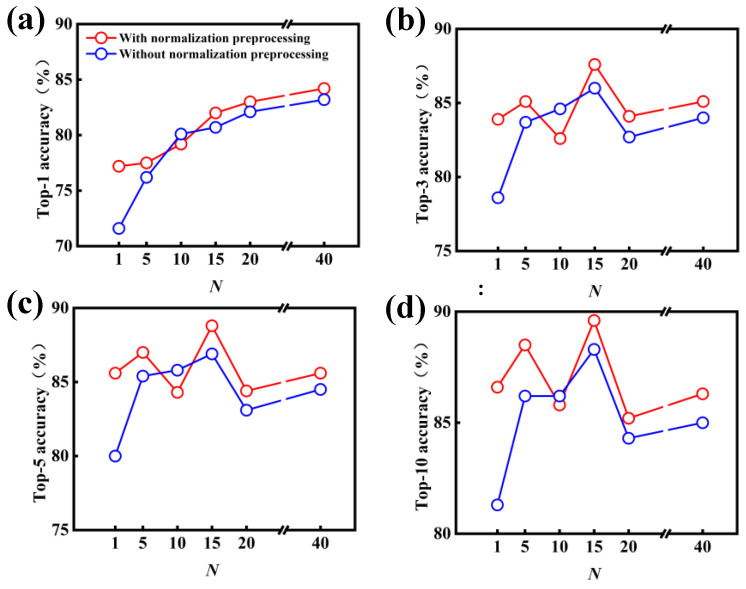
Effect of *N*-level data augmentation on the model performance for the predictions of forward chemical reactions. (**a**) Top-1, (**b**) top-3, (**c**) top-5 and (**d**) top-10 accuracies of the data-driven model with and without normalization preprocessing.

Figure 4 shows the effect of *N*-level data augmentation on the performance of the data-driven model with and without the normalization preprocessing. One can importantly deduce that the introduction of data augmentation results in a significant improvement in the model performance, in comparison with the original dataset of USPTO-50K (i.e., 1-level). In particular, the top-1 accuracy increases from 71.6% to 76.2% as the 5-level data augmentation is applied to the original dataset of USPTO-50K without the normalization preprocessing. With 10-level data augmentation, the top-1 accuracy continues to be improved, reaching 80.1%. As the levels of data augmentation are further increased, the top-1 accuracy can reach 83.2% at the 40-level data augmentation, but the improvement magnitude is no longer as obvious as that of 5- and 10-level data augmentation. Similarly, in comparison with the original dataset, the top-3, top-5 and top-10 accuracies can be improved after the introduction of data augmentation into the data preprocessing. 

Another important outcome is the better performance of the molecular transformer model, which is identified through the introduction of normalization preprocessing; this is shown in Figure 4. In most cases, the accuracy of the data-driven model with normalization preprocessing is better than that without normalization preprocessing. For example, the top-1, top-3, top-5 and top-10 accuracies noticeably increase ~5.0% for the 1-level data augmentation. As the normalization preprocessing is applied to the treatment of the SMILES strings, the accuracies of the data-driven model are increased by different degrees. In particular, the top-1 accuracy can reach 84.2% at the 40-level data augmentation and normalization preprocessing.

**Figure 5:**
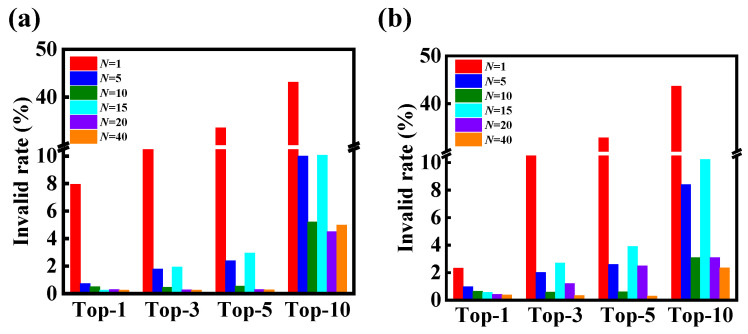
Comparison of invalid rates in terms of the top-1, top-3, top-5 and top-10 accuracies for the model (**a**) without and (**b**) with normalization preprocessing under different *N*-levels of data augmentation. For the sake of clarity, a break is shown in the *y* axis.

To understand why a better performance is observed for the data augmentation and normalization preprocessing, we analyze the incorrect predictions of the molecular transformer model. Considering the easily quantifiable count of grammatically invalid results output by the SMILES strings, we only evaluate the grammatically invalid rate for the output of the molecular transformer model. Figure 5 shows the comparison of invalid rates in terms of the top-1, top-3, top-5 and top-10 accuracies under different *N*-levels of data augmentation. As expected, the data augmentation significantly reduces the grammatically invalid rate of prediction results. For example, the grammatically invalid rate of top-1 accuracy has a value of 7.95% (Figure 5a). When the SMILES enumeration preprocessing is incorporated into the data-driven model, the grammatically invalid rate of top-1 accuracy notably decreases to 0.72% with the 5-level data augmentation, and to 0.24% with the 40-level data augmentation. Similar observations are also identified in the cases of the top-3, top-5 and top-10 accuracies. Namely, the grammatically invalid rate continues to decline with an increase in the number of augmented SMILES strings.

Figure 5a,b also show the invalid rates of the data-driven model without and with normalization preprocessing, respectively. Similar to the case of data augmentation, the grammatically invalid rate is generally reduced by an introduction of normalization preprocessing into the molecular transformer model. In particular, the grammatically invalid rate of top-1 accuracy notably reduces from 7.95% to 2.32% after the introduction of normalization preprocessing. Because the invalid rate is very low with the high-level data augmentation, the impact of normalization preprocessing becomes weak. Taken together, these results obtained from Figure 4 and Figure 5 suggest that the introduction of data augmentation and normalization preprocessing can be harnessed to efficiently improve the performance of the molecular transformer model for the prediction of forward chemical reactions, originating from the reduction in grammatically invalid outputs.

**Figure 6:**
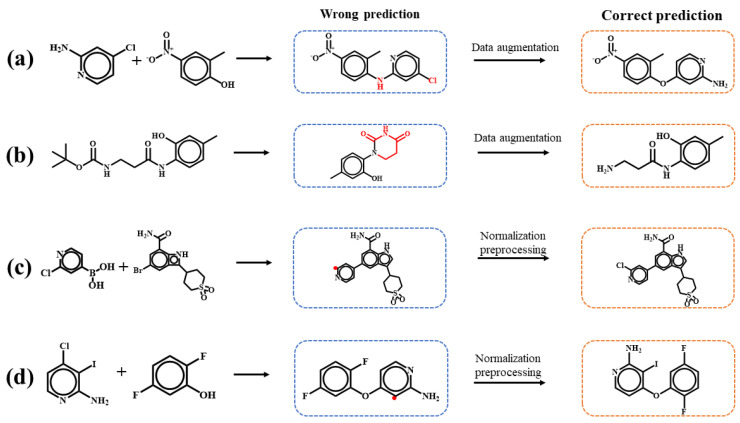
Examples of the predictions of forward chemical reactions from the molecular transformer model with and without (**a**,**b**) the data augmentation and (**c**,**d**) normalization preprocessing. (**a**,**d**) Heteroatom alkylation and arylation reaction, (**b**) deprotection reaction and (**c**) C–C bond formation reaction. The molecular transformer model with (without) the data augmentation and normalization preprocessing predicts the correct (incorrect) products enclosed by the orange (blue) boxes.

Figure 6 illustrates examples of the chemical reactions predicted by the molecular transformer model with and without the data augmentation and normalization preprocessing. Figure 6a,b show the predictions of a heteroatom alkylation and arylation reaction, as well as a simple deprotection reaction for the case of data augmentation, respectively. The original molecular transformer model predicts chemically unreasonable products. After applying the data augmentation strategy, the improved model is able to successfully predict the correct products. Figure 6c,d show the predictions of a C–C bond formation reaction, as well as a heteroatom alkylation and arylation reaction for the case of normalization preprocessing, respectively. Similarly, the introduction of normalization preprocessing into the data-driven model results in the correct predictions of products. 

The findings identified above demonstrate the powerful ability of the model to represent a reaction with multiple SMILES strings and obtains additional information about chemistry from the augmented training data. As a result, the molecular transformer model can significantly reduce the grammatically invalid rate and achieve a high accuracy for the forward reaction predictions. Furthermore, the normalization preprocessing improves the performance of the molecular transformer model, which also originates from the correction of the grammatical errors in the SMILES strings. 

### 3.2. Model Performance on Single-Step Retrosynthesis without Reaction Classes

**Figure 7:**
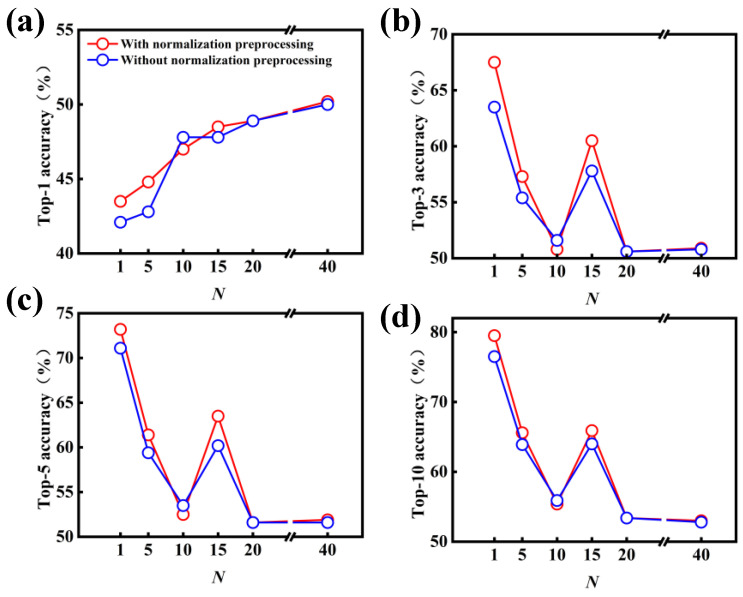
Effect of *N*-level data augmentation on the performance of the data-driven model for the predictions of single-step retrosynthesis without reaction classes. (**a**) Top-1, (**b**) top-3, (**c**) top-5 and (**d**) top-10 accuracies of the model with and without normalization preprocessing.

In the task of single-step retrosynthesis, the input and output of the molecular transformer model are the products and reactants, respectively. Note that the reaction classes are not included in the input of the data-driven model. Figure 7 shows the effect of the *N*-level data augmentation on the performance of the model with and without the normalization preprocessing. Similar to the forward reaction predictions, the top-1 accuracy of the molecular transformer model is improved by the introduction of data augmentation and normalization preprocessing. In particular, the top-1 accuracy can achieve 50.2% for the 40-level data augmentation. However, with the introduction of the data augmentation, the top-3, top-5 and top-10 accuracies decrease, arising from the diversity of the predicted molecules in the task of retrosynthesis.

To further elucidate the trend, we can plot the top-*X* (*X* = 1, 3, 5 and 10) accuracies of the model before and after normalization preprocessing under various levels of data augmentation (Figure S1 of Supplementary Materials). Regardless of the *N*-level of data augmentation, the top-*X* accuracy generally improves with an increase in *X*. It should be pointed out that the performance of the model becomes saturated as the *N*-level of data augmentation is increased.

**Figure 8:**
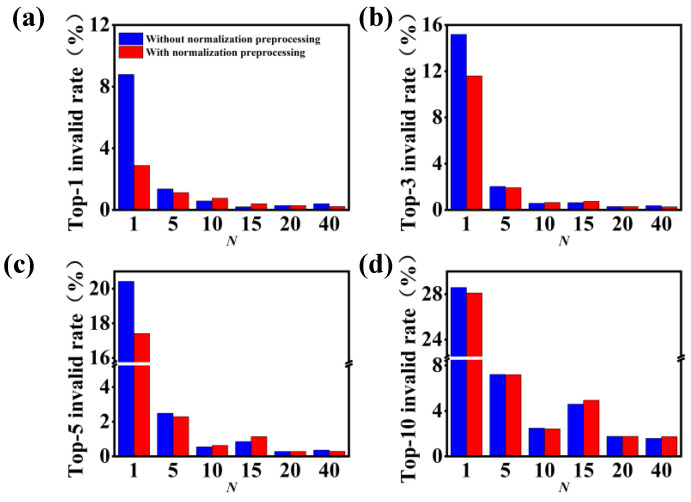
Effect of *N*-level data augmentation on the grammatically invalid rates. (**a**) Top-1, (**b**) top-3, (**c**) top-5 and (**d**) top-10 accuracies of the data-driven model with and without normalization preprocessing.

Figure 8 shows the effect of *N*-level data augmentation on the grammatically invalid rates. As expected, the grammatically invalid rate continues to decrease with an increase in the number of augmented SMILES and the introduction of normalization preprocessing. For example, for the original dataset of USPTO-50K, the grammatically invalid rate of top-1 accuracy reduces from 8.78% to 2.88% after the introduction of normalization preprocessing into the data-driven model. As the 40-level data augmentation is applied to the model, the grammatically invalid rate of top-1 accuracy reduces to 0.22%, corresponding to the higher performance of the molecular transformer model for the task of single-step retrosynthesis. 

**Figure 9:**
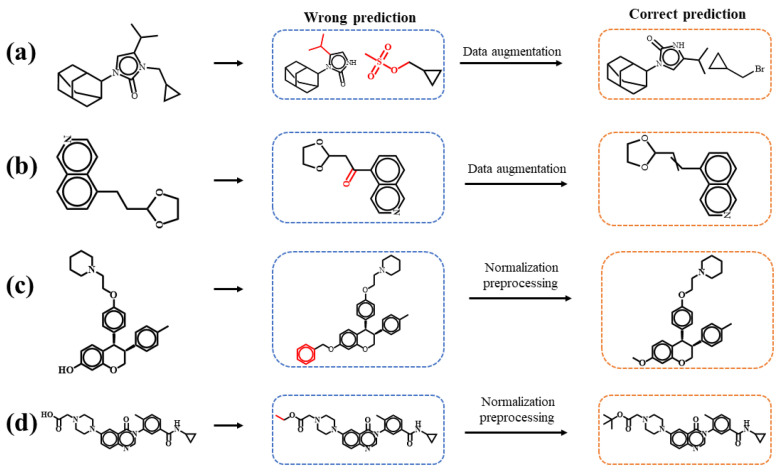
Examples of the predictions of single-step retrosynthesis from the molecular transformer model with and without (**a**,**b**) the data augmentation and (**c**,**d**) normalization preprocessing. (**a**) Substitution reaction, (**b**) reduction reaction, (**c**) functional group interconversion reaction, and (**d**) heteroatom alkylation and arylation reaction. The molecular transformer model with (without) the data augmentation and normalization preprocessing predicts the correct (incorrect) reactants enclosed by the orange (blue) boxes.

Figure 9 illustrates examples of the predictions of single-step retrosynthesis produced by the molecular transformer model with and without the data augmentation and normalization preprocessing. For the simple substitution reaction and reduction reaction (Figure 9a,b), the molecular transformer model for the original dataset of USPTO-50K predicts chemically unreasonable reactants. After applying the data augmentation strategy to data preprocessing, the data-driven model successfully predicts the correct reactants. Similarly, the introduction of normalization preprocessing results in the correct prediction of reactants for the functional group interconversion, as well as the heteroatom alkylation and arylation reactions (Figure 9c,d).

### 3.3. Model Performance on Single-Step Retrosynthesis with Reaction Classes

**Figure 10:**
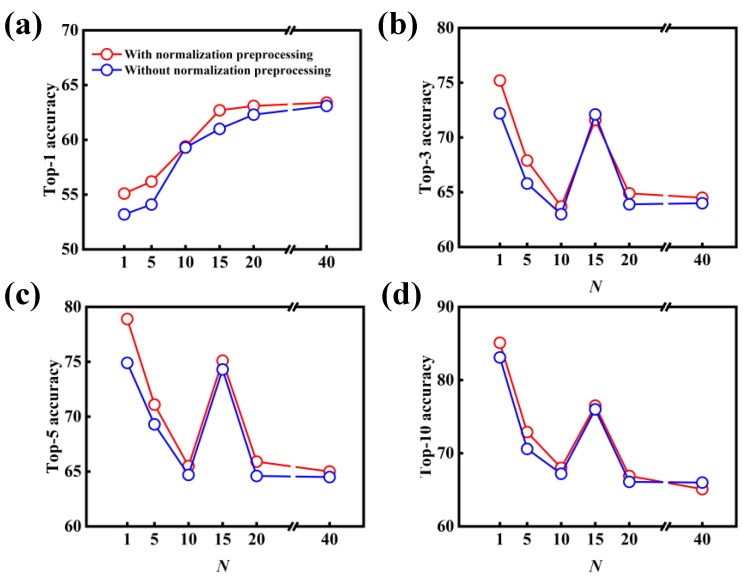
Effect of *N*-level data augmentation on the performance of model for the prediction of single-step retrosynthesis with reaction classes. (**a**) Top-1, (**b**) top-3, (**c**) top-5 and (**d**) top-10 accuracies of the model with and without normalization preprocessing.

Unlike the task carried out in Section 3.2, the input of the molecular transformer model includes the products and the reaction classes, which are listed in Figure 1. Namely, more information about the chemical reactions is introduced to the data-driven model for the prediction of retrosynthesis. Figure 10 shows the effect of *N*-level data augmentation on the performance of the model with reaction classes. Similar to the results of the model without the reaction classes, the top-1 accuracy of the molecular transformer model is improved by the introduction of data augmentation and the normalization preprocessing. It is of note that, because the additional information of the chemical class is introduced to the input of the data-driven model, the prediction accuracies of the molecular transformer model are higher than when the reaction classes are absent. In particular, with the introduction of data augmentation and the normalization preprocessing, the top-1 accuracy can achieve a higher value of 63.4%, which is currently the best performance for single-step retrosynthesis.

In addition, a significant conclusion may be drawn from the observation of Figure 7 and Figure 10. Namely, with the 15-level data augmentation, the model shows excellent performance. When the amount of data augmentation is low, such as 5-level or 10-level data augmentation, the performance of the model is not noticeably enhanced due to the limited availability of the database. The model learns to predict several representations of the same molecule when trained on a high number of augmented SMILES, such as 20-level and 40-level data augmentation. The model predicts many SMILES strings for the same molecule in various ways. Therefore, the model with 15-level data augmentation shows excellent performance.

**Figure 11:**
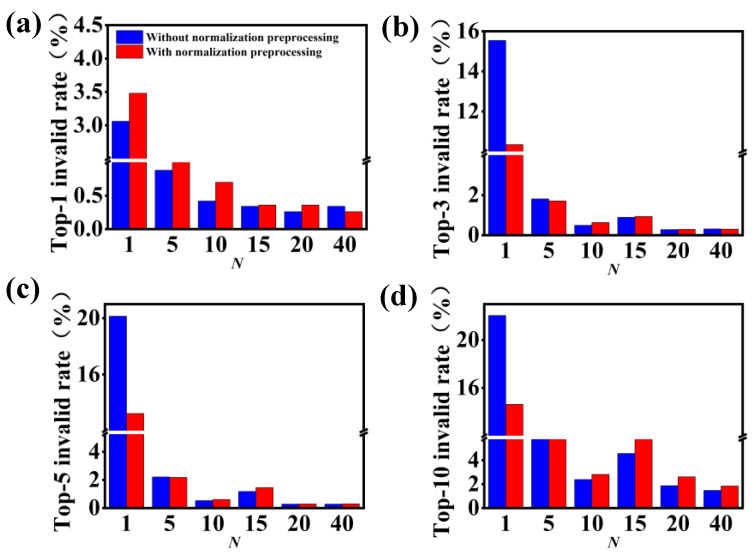
Effect of *N*-level data augmentation on the grammatically invalid rates for the retrosynthetic prediction with additional reaction classes as the input. (**a**) Top-1, (**b**) top-3, (**c**) top-5 and (**d**) top-10 accuracies of the model with and without normalization preprocessing.

Figure 11 shows the impacts of data augmentation and normalization preprocessing on the grammatically invalid rates for the single-step retrosynthetic prediction with additional reaction classes. In general, the grammatically invalid rate decreases with an increase in the *N*-level of data augmentation and the introduction of normalization preprocessing, especially for the case of 5-level data augmentation. In particular, by comparing the grammatically invalid rates in Figure 8 and Figure 11, one can importantly deduce that the chemical classes provide additional information for the achievement of the superior performance of the molecular transformer model, which is applied to the one-to-many task of retrosynthetic predictions. 

**Figure 12:**
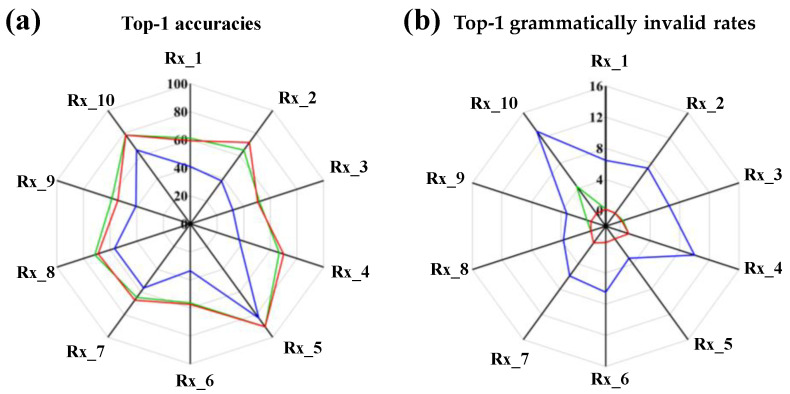
(**a**) Top-1 accuracies and (**b**) grammatically invalid rate in terms of the reaction classes for the single-step retrosynthesis. The blue lines correspond to the original UPSTO-50K dataset without normalization preprocessing. The green and red lines are the 40-level augmented dataset without and with normalization preprocessing, respectively.

In order to further understand the impacts of the data augmentation and normalization preprocess on the performance of the data-driven model, we evaluate the top-1 accuracy and grammatically invalid rate in terms of the reaction class, which are shown in Figure 12. The distributions of the reaction classes within the USPTO-50K are depicted in Figure 1. The reaction classes Rx_1 (heteroatom alkylation and arylation), Rx_2 (acylation and related processes) and Rx_6 (deprotections) have a large number of items, but the items of Rx_4 (heterocycle formation), Rx_5 (protections), Rx_8 (oxidations) and Rx_10 (functional group addition) are extremely scarce. A common outcome is identified in Figure 12. For all the reaction classes, the top-1 accuracies are significantly improved and correspondingly, the invalid rates are reduced via the data augmentation and normalization preprocess in the molecular transformer model. 

More importantly, from the detailed observations in Figure 12, one can deduce another important outcome. The accuracies of Rx_1, Rx_2 and Rx_6, with a large number of reaction items, are relatively low. The most significant improvement in accuracy for the three reaction classes is observed due to our strategies of data augmentation and normalization preprocessing (Figure 12a). Meanwhile, the scarce Rx_4, Rx_5, Rx_8 and Rx_10 reaction classes have relatively high accuracies, which can be also promoted by our proposed strategies. However, a different trend is also observed regarding the change in the invalid rates associated with the proposed strategies. As shown in Figure 12b, the Rx_4, Rx_5, Rx_8 and Rx_10, which have a relatively small number of reaction items, have relatively high invalid rates. After the introduction of our data augmentation and normalization preprocessing strategies, their invalid rates significantly decreased.

The findings illustrated above prove the powerful ability of the proposed model to represent a reaction using multiple SMILES strings and normalization preprocessing in order to predict the outcome of retrosynthesis. Our proposed strategies for dataset preprocessing are able to reduce the grammatically invalid rates and improve the accuracy of the molecular transformer model. For the reaction classes with a large number of items, our proposed strategies are able to significantly improve their prediction accuracy. For the scarce reaction classes, the proposed strategies are able to significantly reduce their grammatically invalid rate.

## 4. Conclusions

In this work, by virtue of the strategies of data augmentation and normalization preprocessing, we use the molecular transformer model to accomplish the three tasks of chemical reactions (i.e., the forward predictions of chemical reactions, and single-step retrosynthetic predictions without and with the reaction classes). It is found that the performance of such a data-driven model can be significantly improved by our proposed strategies for the three tasks of chemical reactions. In particular, the top-1 accuracies of forward and single-step retrosynthetic reaction predictions can, respectively, achieve higher values of 84.2% and 63.4% with 40-level data augmentation and normalization preprocessing, which are currently the best performance results for the tasks of chemical reactions. It is clearly demonstrated that the superior performance of the data-driven model originates from the correction of grammatical errors in the SMILES strings, especially for the case of the scarce reaction classes. The data augmentation and normalization preprocessing proposed in this study provide useful strategies by which to improve the prediction capabilities of chemical reactions with a small dataset. We anticipate that these strategies will be integrated into other machine learning models in order to further accelerate this AI-assisted retrosynthetic route in the fields of organic chemistry and polymer chemistry. 

## Data Availability

The code and the data presented in this study are available upon reasonable request from the corresponding authors.

## Supplementary Materials

The following supporting information can be downloaded at: https://www.mdpi.com/article/10.3390/polym15092224/s1, Figure S1: The Top-X accuracies of the model before and after normalization preprocessing at (a)1-, (b) 5-, (c) 10-, (d) 15-, (e) 20-, and (f) 40-level data augmentation..
