# Supplementary material for: Harnessing Data Augmentation and Normalization Preprocessing to Improve the Performance of Chemical Reaction Predictions of Data-Driven Model
Source: Polymers (Basel). 2023 May 8;15(9):2224. doi: 10.3390/polym15092224 (PMC10180765; doi:10.3390/polym15092224)
Supplement: Supplementary file 1 [file polymers-15-02224-s001.zip › polymers-2347835-supplementary.pdf]

Supplementary

Harnessing Data Augmentation and Normalization Preprocessing to Improve the Performance of Chemical Reaction Predictions of Data-Driven Model

Boyu Zhang, Jiaping Lin *, Lei Du and Liangshun Zhang *

Shanghai Key Laboratory of Advanced Polymeric Materials, School of Materials Science and Engineering, East China University of Science and Technology, Shanghai 200237, China

* Correspondence: jlin@ecust.edu.cn (J.L.); zhangls@ecust.edu.cn (L.Z.)

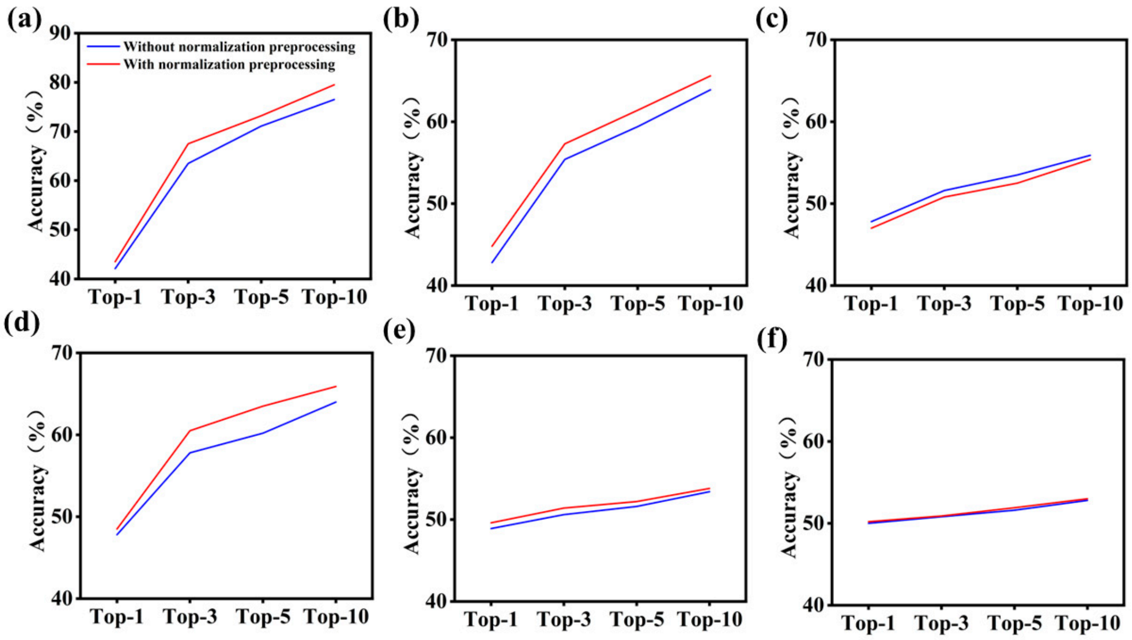

Figure S1. The Top-X accuracies of the model before and after normalization preprocessing at (a)1-, (b) 5-, (c) 10-, (d) 15-, (e) 20-, and (f) 40-level data augmentation.
